# Dual Transverse Arch Foot Orthosis Improves Gait Biomechanics in Females with Flexible Flatfoot

**DOI:** 10.3390/bioengineering12040418

**Published:** 2025-04-14

**Authors:** Linjie Zhang, Qiaolin Zhang, Qian Liu, Xinyan Jiang, János Simon, Tibor Hortobágyi, Yaodong Gu

**Affiliations:** 1 Department of Radiology, Ningbo No. 2 Hospital, Ningbo 315010, China; 2 Department of Kinesiology, Hungarian University of Sports Science, 1123 Budapest, Hungary; 3 Doctoral School of Safety and Security Sciences, Óbuda University, 1034 Budapest, Hungary; 4 Faculty of Engineering, University of Szeged, 6720 Szeged, Hungary; 5 Institute of Sport Sciences and Physical Education, Faculty of Sciences, University of Pécs, 7622 Pécs, Hungary; 6 Department of Human Movement Sciences, University Medical Center Groningen, University of Groningen, 9712 Groningen, The Netherlands; 7 Faculty of Sport Science, Ningbo University, Ningbo 315211, China

**Keywords:** flexible flatfoot, foot orthoses, transverse arch support, medial longitudinal arch, gait analysis, joint kinematics, foot stability

## Abstract

(1) Background: Flexible flatfoot is characterized by medial arch collapse, leading to musculoskeletal impairments. We examined the effects of single-arch foot orthosis (SFO) and dual-arch foot orthosis (DFO) on arch height, kinematics, and kinetics in young females during walking and jogging. (2) Methods: Healthy females (n = 19) with flexible flatfoot were tested under three conditions: regular shoes, SFO, and DFO. Motion capture and a 3D force plate gathered biomechanical data. We also used a high-speed dual fluoroscopic imaging system (DFIS) to assess dynamic foot morphology. Outcomes included normalized truncated navicular height, medial arch angle, angles and moments at the metatarsophalangeal, subtalar, ankle, knee, and hip joints. (3) Results: Both types of orthoses improved the normalized navicular height and reduced the medial arch angle, with DFO vs. SFO showing greater effects (*p* < 0.001). DFO vs. SFO was also more effective in limiting the range of motion (ROM) of the metatarsophalangeal joint and dorsiflexion (*p* < 0.001). Additionally, DFO reduced the ankle range of motion and the maximum knee flexion during walking. Both orthoses reduced subtalar plantarflexion moments during stance (*p* < 0.001) and modulated ankle plantarflexion moments throughout different phases of gait. DFO uniquely enhanced metatarsophalangeal plantarflexion moments during jogging (*p* < 0.001). (4) Conclusions: Dual vs. single transverse arch foot orthosis is more effective in improving gait biomechanics in females with flexible flatfoot. Longitudinal studies are needed to confirm these benefits.

## 1. Introduction

The human foot has evolved to be highly flexible to accommodate stiffness demands during ambulatory and jumping activities [1,2,3]. The two longitudinal arches medial and lateral, and the transverse arch are important determinants of foot stiffness [4,5]. The medial longitudinal arch is particularly important for maintaining midfoot stiffness and providing elastic responses to perturbations in the sagittal plane. This function is supported by the bowstring arrangement of the plantar fascia [1] and the windlass mechanism initiated by dorsiflexion in the metatarsophalangeal joint [6]. Consequently, structural defects in the medial longitudinal arch can impair foot function, resulting in flatfoot, a common musculoskeletal disorder [7]. Flatfoot can be categorized as either flexible or rigid, depending on the arch’s response to weight bearing. Flexible flatfoot, the most prevalent type, is characterized by medial longitudinal arch collapse only under load [8]. It is often accompanied by structural changes such as talonavicular inversion, heel valgus, midfoot inversion, and forefoot abduction/pronation [9]. Such aberrations disrupt normal posture and movement mechanics, increasing the risk of foot injuries and pain [10,11]. Common complications of flatfoot include plantar fasciitis, plantar fascia pain, foot instability, ankle pain, and ligamentous laxity [12,13,14]. Abnormal foot stiffness and altered load distribution can lead to metatarsal stress fractures and knee osteoarthritis [14,15,16]. Moreover, foot instability can impact proximal joints, such as the ankle and hip, and may extend to the spine through compensatory mechanisms, resulting in conditions like medial tibial stress syndrome [17], pelvic tilt [18], lumbar lordosis and low back pain [19,20]. These complications can significantly diminish the patient’s quality of life, limit physical activity, and hinder social participation [21].

Foot orthoses (FOs) are commonly used medical devices for treating flexible flatfoot [22,23]. FOs are designed to support the medial longitudinal arch, limit hindfoot range of motion, and redistribute plantar pressure to correct alignment and improve clinical symptoms while preventing disease progression [24]. Biomechanical studies suggest that FOs can favorably modify foot alignment and posture, reduce subtalar and ankle overpronation [25], prevent medial longitudinal arch collapse, and inhibit excessive foot elongation [26]. FOs have also been reported to decrease peak ankle plantarflexion and dorsiflexion angles [27,28], reduce the second peak of vertical ground reaction forces during walking [27], and diminish peak ankle valgus moment and power absorption [29,30]. However, the effects of FOs on foot kinematics and dynamics remain inconclusive. For example, Cheng et al. [9] observed that 3D-printed FOs increased peak ankle dorsiflexion compared to standard footwear, while Karimi et al. [27] found no significant effects of FOs on ankle range of motion. Similarly, studies by Hurd et al. [31] and Zifchock et al. [32] reported no significant reductions in hindfoot valgus with FO use. These conflicting results may stem from differences in methods between studies, sample characteristics, and FO designs.

Recent research highlights the importance of the transverse arch in maintaining foot transverse arch stability. Venkadesan et al. [5] demonstrated that the transverse arch contributes more than 40% to overall foot stiffness, while Tawar et al. [33] showed that increasing transverse arch stiffness through bandaging resulted in a 53% increase in foot stiffness. Using finite element modeling suggests that doubling forefoot transverse arch stiffness increased longitudinal stiffness by 33%, reduced navicular drop by 54%, and minimized foot elongation by ~3% [34]. Krüger et al. [35], using cadaveric experiments, found that weakness of the transverse tarsal arch significantly influenced progressive foot collapse. Transverse arch stability seems to be closely related to flatfoot pathology. The question is whether supporting the transverse arch could reduce complications caused by flatfoot and arch collapse. Currently, there is a lack of research on the biomechanical effects of dual-arch foot orthosis (DFO) with transverse arch support in patients with flatfoot.

This study aims to compare the sagittal plane biomechanics of lower limb joints during walking and jogging in females with flexible flat feet while wearing three types of footwear: regular shoes, single-arch foot orthosis (SFO), or DFO. Additionally, high-speed dual fluoroscopy, a dynamic radiographic imaging system, can reveal foot morphology in motion. We hypothesized that (1) both SFO and DFO would significantly alter sagittal plane kinematics by reducing metatarsophalangeal and ankle dorsiflexion ranges and increasing subtalar dorsiflexion angles, with greater effects from DFO; (2) both SFO and DFO would significantly affect sagittal plane kinetics by decreasing subtalar plantarflexion moments and modulating ankle joint moments, with DFO demonstrating greater effectiveness; (3) dynamic radiographic analysis would demonstrate that both orthoses effectively enhance foot structural stability—evidenced by increased navicular height and decreased medial arch angle—with greater effects produced by DFO vs. SFO. Our findings contribute to a deeper understanding of the biomechanical effects of FOs and provide new perspectives and theoretical support for the clinical management of flatfoot.

## 2. Materials and Methods

### 2.1. Subject Recruitment

The required sample size was calculated using G*Power 3.1 [36], based on a bilateral design with an effect size (dz) of 0.8, an alpha level of 0.05, and a power (1-β) of 0.8, resulting in a minimum of 15 participants. We recruited 23 volunteers through social media, email, posters, and personal invitations on campus. We confined the sample to females to reduce variability in foot biomechanics [37]. Before enrollment, all participants received detailed information about the study, including its purpose, procedures, and potential risks. Each participant signed an informed consent that was approved by the University Ethics Committee (approval number: RAGH20240610).

Flexible flatfoot was confirmed by measuring the resting calcaneal stance position angle (≥4° valgus) with a standard goniometer [38]. To reduce variation caused by between-leg differences in foot morphology, we collected data in the right-dominant leg. After screening, 19 participants met the inclusion criteria as follows: (1) female, aged 18–40 years; (2) no lower extremity injury within the last six months; (3) no history of lower extremity or spinal surgery; (4) no musculoskeletal or neurological disorders affecting movement; (5) flexible flatfoot; and (6) right-leg dominance, defined as the preferred leg for kicking; (7) no radiological imaging examinations have been performed in the past year, and (8) not pregnant. All experiments were conducted in the Biomechanics Laboratory.

### 2.2. Foot Orthoses

**Figure 1:**
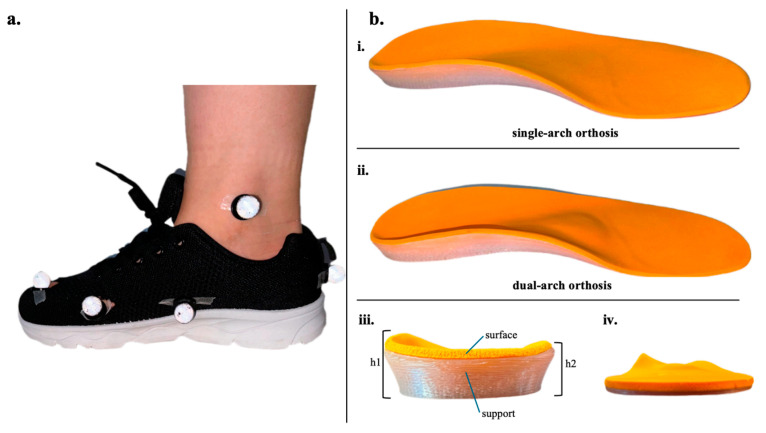
(**a**) Running shoe with reflective markers attached to anatomical landmarks for 3D motion capture analysis. Each subject wore the same style of shoes provided by laboratory staff. (**b**) Foot orthoses conditions: (i) Single-arch foot orthosis (SFO), providing medial longitudinal arch support only. (ii) Dual-arch foot orthosis (DFO), providing combined support for the medial longitudinal arch and transverse arch (mild hump in the middle of the insert). (iii) Rear view of orthosis illustrating support structure (h1: inner support height, h2: outer lateral height). (iv) Medial-side perspective demonstrating orthosis contour and arch support design.

The experimental footwear consisted of standardized commercially available running shoes (Figure 1a). Two types of FOs were used in the experiment: a SFO providing medial longitudinal arch support, and a DFO offering both medial longitudinal arch and transverse arch support as illustrated in Figure 1b. Aside from the addition of transverse arch support in the DFO (Figure 1b), all other design parameters remained consistent between the two orthoses (Table 1). The laboratory customized two types of FOs for each size according to the size of the subject’s foot.

### 2.3. Experimental Design

**Figure 2:**
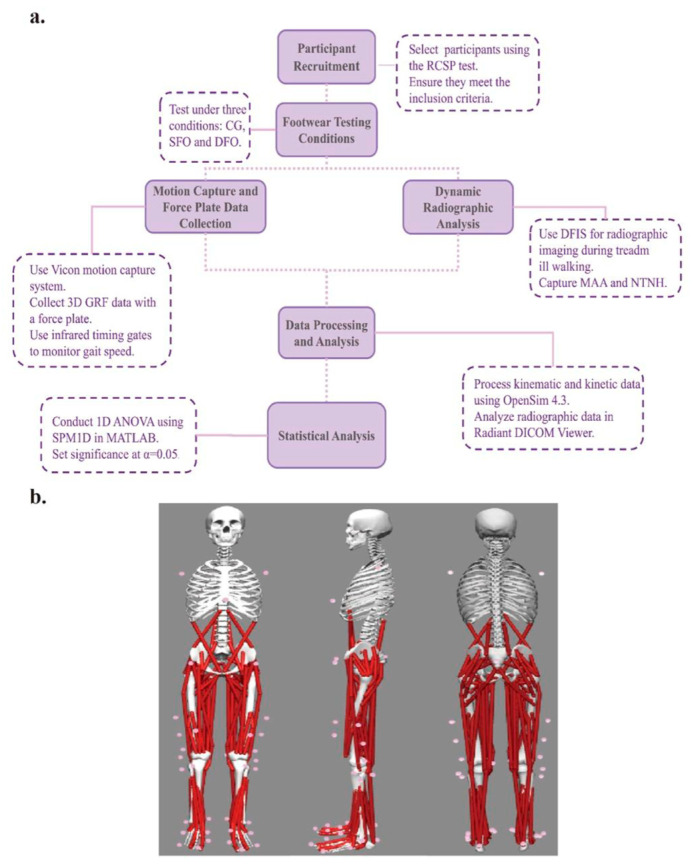
(**a**) Experimental design and procedures. (**b**) The marked front, side, and back views with pink-colored markers. CG: control group; SFO: single-arch foot orthosis; DFO: dual-arch foot orthosis; MAA: medial arch angle; NTNH: normalized truncated navicular height; vGRF: vertical ground reaction force; DFIS: dual fluoroscopic imaging system; RCSP: resting calcaneal stance position; SPM1D: one-dimensional statistical parametric mapping.

To ensure that the observed differences were due to the orthotic condition rather than inherent characteristics of the participants, a randomized crossover design was used in this study, with each participant performing the test conditions (regular shoes, SFO, DFO) in a random order to minimize potential order effects. The control group (CG) wore standard running shoes without orthotic inserts. Anthropometric measurements, including height and weight, were collected for all participants. The experimental process is shown in Figure 2a.

#### 2.3.1. Kinematic and Kinetic Data Collection

A motion capture system consisting of eight infrared cameras (Vicon Metrics Ltd., Oxford, UK) was used to record movement trajectories at a frequency of 200 Hz. Simultaneously, a 3D force plate (AMTI, Watertown, MA, USA) collected kinetic data at 1000 Hz. Infrared timing gates (Brower Timing System, Draper, UT, USA) were placed 1.8 m apart at both ends of the force plate to calculate gait speed by measuring the time taken to pass through these points. Participants were instructed to test three footwear conditions—regular running shoes, SFO with regular running shoes, and DFO with regular running shoes at two self-selected speeds (walking and jogging). The testing order of the footwear conditions was randomized.

Before the formal experiment, participants performed three practice trials in regular shoes for each speed to determine their average self-selected speed, which served as the baseline. A ±5% speed variation was permitted during testing. Before the formal tests, all participants were given 15 min to warm up and familiarize themselves with the experimental setup. Standardized compression shirts and tights were worn during testing. A total of 38 reflective markers were strategically placed based on the Gait 2392 musculoskeletal model (as shown in Figure 2b). After marker placement, participants stood on the force plate in a standard anatomical position for static data collection. During dynamic trials, participants walked or ran along a 10 m track with the force plate positioned in the center. A successful trial required the participant’s right foot to make full contact with the force plate during the support phase without stepping outside its boundaries. Each speed condition required eight successful trials per footwear type. A valid trial was defined as one where the heel made initial contact and toe-off occurred within the plate boundary, with the vertical ground reaction force (vGRF) exceeding 10 N during initial contact [39]. A 30 s rest interval was provided between trials to ensure participants had adequate time for recovery.

#### 2.3.2. Radiographic Analysis

**Figure 3:**
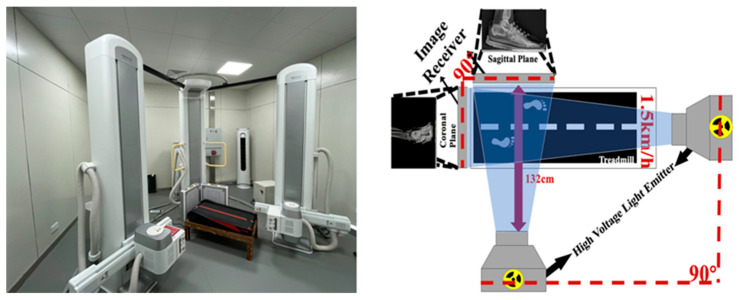
Schematic diagram of DFIS equipment and radiological image acquisition.

Dynamic foot radiographs were captured using a dual fluoroscopic imaging system (DFIS), which consisted of two pairs of X-ray emitters and two image intensifiers arranged perpendicularly to capture both coronal and sagittal plane views. DFIS consists of a motion perspective system and a data analysis system. The motion perspective system consists of two high-voltage light transmitters and two image receivers. The distance between the two high-voltage light transmitters and the image receivers is 132 cm, and the angle between the image receivers is 90° [40]. The subject walked on the treadmill at 1.5 m/s, with a transmission voltage of 60 kV, a current of 63 mA, and a transmission frequency of 60 Hz (Figure 3). Before testing, participants were given 15 min to warm up and acclimate to the setup. During testing, participants walked on a treadmill at a speed of 1.5 m/s under three randomized conditions: regular running shoes, SFO with regular running shoes, and DFO with regular running shoes. The single run time is from DFIS preparation to the end of shooting, about 30 s. Each condition was assessed with one successful trial using the DFIS, and image quality was used as the criterion for trial selection [39]. Professional supervision was provided throughout the process to ensure clear imaging of the right foot during trials.

### 2.4. Data Processing

Kinematic data and ground reaction forces collected by the Vicon system were exported in C3D format files. MATLAB R2017b (The MathWorks, Natick, MA, USA) was used to perform coordinate transformations, low-pass filtering, data extraction, and format conversion. Biomechanical parameters were calculated using OpenSim 4.3 (Stanford University, Stanford, CA, USA) with the Gait2392 musculoskeletal model. The model was scaled for each participant based on marker positions and body weight to determine accurate muscle origins and insertions, ensuring consistency in limb lengths [41]. The scaled model was then used in the Inverse Kinematics and Inverse Dynamics tools in OpenSim to calculate joint angles and moments.

**Figure 4:**
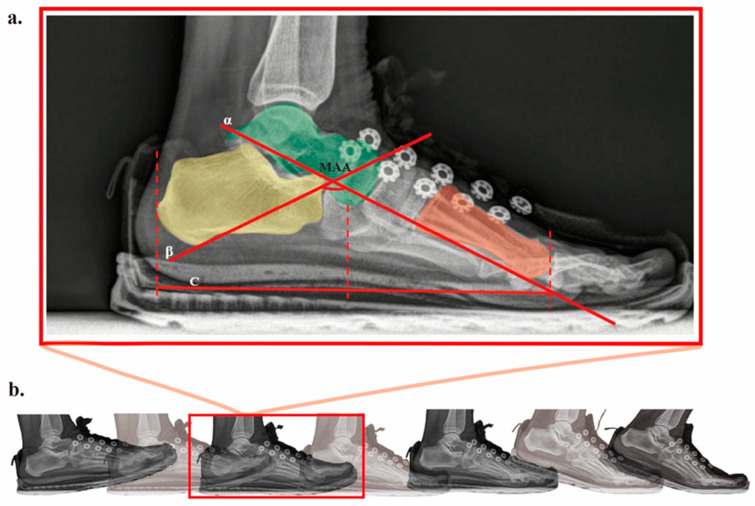
(**a**) NTNH: the distance from the lowest point of the navicular bone to line segment C divided by the truncated foot length (line segment C); MAA: the obtuse angle between line α and line β. (**b**) Foot images at different stages of the stance phase.

Dynamic foot radiographic data captured by the DFIS were imported into Radiant DICOM Viewer (Medixant, Poznan, Poland) in DCOM format. To minimize measurement errors, three experienced radiologists independently evaluated the parameters for each frame, and the average values were recorded. As shown in Figure 4a, the measured parameters included normalized truncated navicular height (NTNH) and medial arch angle (MAA). NTNH was defined as the navicular height divided by the truncated foot length (excluding the toes) [42,43], and MAA was defined as the angle between the line connecting the lowest point of the sesamoid bone to the lowest point of the talonavicular joint and the line from the lowest point of the subtalar joint to the lowest point of the calcaneus (Figure 4a) [44]. Time normalization of joint kinematics within the stance phase was achieved using interpolation, standardizing the execution time of lower limb joint angles, joint moments, vertical ground reaction forces, normalized truncated navicular height, and medial arch angle. Figure 4b shows radiographic images of the foot at different times during the support phase of gait.

### 2.5. Statistical Analysis

Statistical analysis was conducted using SPSS version 26.0 (IBM, Armonk, NY, USA). Descriptive statistics, including mean and standard deviation (SD), were calculated. Normality was verified using the Shapiro–Wilk test, and all data met the normal distribution criteria. Given the one-dimensional time-varying nature of joint kinematics and kinetics, one-way ANOVA was conducted using one-dimensional statistical parametric mapping, with post hoc tests using Tukey HSD. All statistical analyses were performed in MATLAB R2017a (The MathWorks, Natick, MA, USA), with a significance level set at α = 0.05, and *p*-values < 0.05 were considered statistically significant.

## 3. Results

Table 2 shows the descriptive data for the 19 participants who met the inclusion criteria.

### 3.1. Kinematics

#### 3.1.1. Normalized Truncated Navicular Height (NTNH)

**Figure 5:**
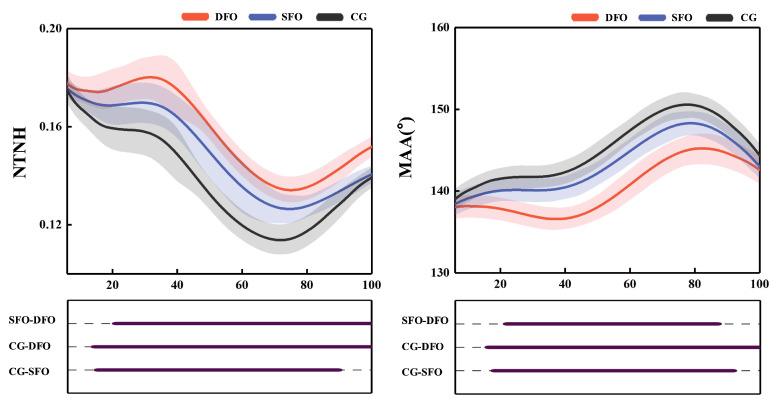
Changes in NTNH and MAA with different FOs during the stance phase from SPM. DFO represents the condition of wearing shoes with double arch support orthosis, SFO represents the condition of wearing shoes with single arch support orthosis, and CG represents the control condition of not wearing any foot orthosis.

The time-series data for NTNH are shown in Figure 5. Compared to the control condition, the DFO significantly increased NTNH during 9% to 100% of the stance phase (*p* < 0.001), while the SFO showed a significant increase in NTNH from 10.3% to 89% of the stance phase (*p* < 0.001). Compared to SFO, the DFO led to a significant increase in NTNH from 16% to 100% of the stance phase (*p* < 0.001).

Table 3 shows that peak NTNH is higher by 0.02 (95% CI: −0.02, −0.02, *p* < 0.001) in the DFO condition compared to the control and by 0.01 (95% CI: −0.01, −0.01, *p* < 0.001) compared to SFO in the stance phase of gait. Range of motion decreased by 0.02 (95% CI: 0.02, 0.03, *p* < 0.001) compared to the control and by 0.01 (95% CI: 0.01, 0.02, *p* = 0.005) compared to SFO.

#### 3.1.2. Medial Arch Angle (MAA)

The time-series data for MAA are shown in Figure 5. Compared to the SFO and control conditions, the DFO significantly reduced the MAA during 13% to 91% of the stance phase (*p* = 0.001 and *p* < 0.001, respectively). Similarly, the SFO condition significantly decreased the MAA during 13% to 91% of the stance phase compared to the control condition (*p* = 0.026). Table 3 shows that compared to control, the peak MAA was reduced by 4.5° (95% CI: 4.22–4.85, *p* < 0.001) in the DFO condition and by 2.3° (95% CI: 1.96–2.56, *p* < 0.001) in the SFO condition. The DFO condition also reduced the range of motion by 0.7° (95% CI: 0.27–1.14, *p* = 0.003). Furthermore, compared to the SFO condition, the DFO condition showed greater reductions in peak MAA by 2.3° (95% CI: 1.84–2.71, *p* < 0.001).

#### 3.1.3. Lower Limb Position

**Figure 6:**
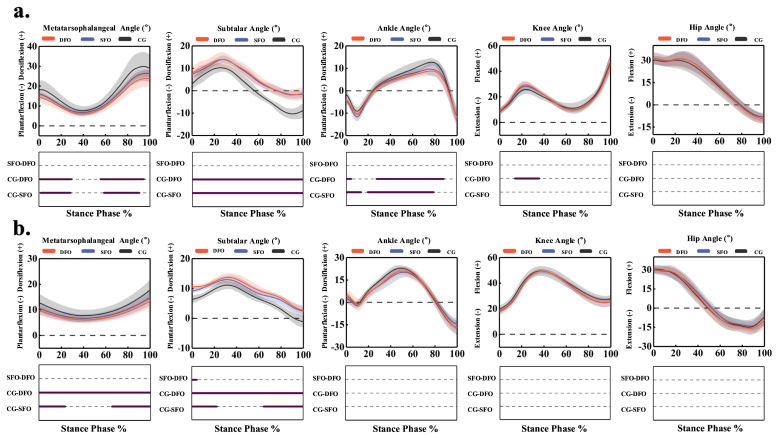
Impact of FOs on lower limb joint angles in the sagittal plane during walking and jogging from SPM. (**a**) Walking. (**b**) Jogging. DFO represents the condition of wearing shoes with double arch support orthosis, SFO represents the condition of wearing shoes with single arch support orthosis, and CG represents the control condition of not wearing any foot orthosis.

The time-series data for joint angles are shown in Figure 6. During walking, compared to the control condition, the SFO condition significantly reduced metatarsophalangeal joint dorsiflexion at 2% to 29.4% (*p* < 0.001) and 60% to 91.5% of the stance phase (*p* < 0.001) while significantly increasing subtalar joint dorsiflexion throughout 0% to 100% of the stance phase (*p* < 0.001). SFO also significantly increased ankle plantarflexion from 0% to 14.4% (*p* < 0.001) and reduced dorsiflexion from 21% to 80.8% of the stance phase (*p* < 0.001).

In the DFO condition, metatarsophalangeal dorsiflexion was significantly reduced at 1% to 30.5% (*p* < 0.001) and 57% to 95.5% (*p* < 0.001) of the stance phase, while subtalar joint dorsiflexion increased significantly throughout 0% to 100% (*p* < 0.001). Ankle plantarflexion significantly increased from 0% to 4.6% (*p* < 0.001), and dorsiflexion decreased significantly from 29.1% to 89.3% of the stance phase (*p* < 0.001). Additionally, knee flexion angle increased significantly during the load response phase from 15% to 36.3% of the stance phase (*p* < 0.001).

During jogging, SFO significantly reduced metatarsophalangeal dorsiflexion at 0% to 24% (*p* < 0.001) and 67.5% to 100% (*p* < 0.001) while increasing subtalar joint dorsiflexion at 0% to 22.5% (*p* < 0.001) and 66% to 100% (*p* < 0.001) of the stance phase. In the DFO condition, metatarsophalangeal joint dorsiflexion was significantly reduced throughout 0% to 100% of the stance phase, while subtalar joint dorsiflexion significantly increased throughout 0% to 100% (*p* < 0.001). Compared to SFO, DFO significantly increased subtalar joint dorsiflexion at 0% to 4.4% (*p* < 0.001). No significant differences in hip joint angles were observed between the conditions at either speed.

As shown in Table 3, during walking, compared to the control condition, both SFO and DFO significantly reduced metatarsophalangeal joint ROM by 2.50° (95% CI: 0.54–4.44, *p* = 0.013) and 4.44° (95% CI: 1.01–6.45, *p* < 0.001), respectively; increased maximum subtalar dorsiflexion by 4.03° (95% CI: 2.89–5.17, *p* < 0.001) and 4.26° (95% CI: 2.02–6.49, *p* = 0.002), respectively; reduced subtalar ROM by 4.79° (95% CI: 3.17–6.41, *p* < 0.001) and 4.17° (95% CI: 2.53–5.79, *p* < 0.001), respectively; and decreased maximum ankle dorsiflexion by 3.59° (95% CI: 1.66–5.51, *p* = 0.002) and 3.98° (95% CI: 2.73–5.22, *p* < 0.001), respectively. Additionally, the DFO significantly reduced maximum metatarsophalangeal dorsiflexion by 6.04° (95% CI: 3.41–8.66, *p* < 0.001), ankle ROM by 3.85° (95% CI: 2.20–5.50, *p* < 0.001), and maximum knee flexion by 2.07° (95% CI: 0.05–4.10, *p* = 0.031). Compared to the SFO condition, the DFO significantly reduced maximum metatarsophalangeal dorsiflexion by 2.61° (95% CI: 1.55–3.67, *p* < 0.001) and ROM by 1.94° (95% CI: 1.14–2.75, *p* < 0.001).

As shown in Table 4, during jogging, compared to the control condition, both SFO and DFO significantly reduced maximum metatarsophalangeal dorsiflexion by 2.97° (95% CI: 1.55–4.38, *p* < 0.001) and 4.43° (95% CI: 2.97–5.89, *p* < 0.001), respectively; reduced ROM by 1.88° (95% CI: 0.54–4.44, *p* = 0.013) and 2.33° (95% CI: 0.60–4.06, *p* < 0.001), respectively; increased maximum subtalar dorsiflexion by 1.61° (95% CI: 0.71–2.56, *p* = 0.040) and 2.52° (95% CI: 1.12–3.92, *p* = 0.020), respectively; and significantly decreased maximum ankle dorsiflexion by 1.82° (95% CI: 0.39–2.38, *p* = 0.004) and 1.90° (95% CI: 0.15–3.65, *p* = 0.036), respectively. Additionally, SFO significantly reduced ankle ROM by 1.91° (95% CI: 0.32–3.50, *p* = 0.021). Compared to the SFO condition, the DFO significantly further reduced maximum metatarsophalangeal dorsiflexion by 1.53° (95% CI: 0.89–2.04, *p* < 0.001) and ROM by 0.79° (95% CI: 0.50–1.14, *p* < 0.001).

### 3.2. Kinetics

#### 3.2.1. Vertical Ground Reaction Force (vGRF)

**Figure 7:**
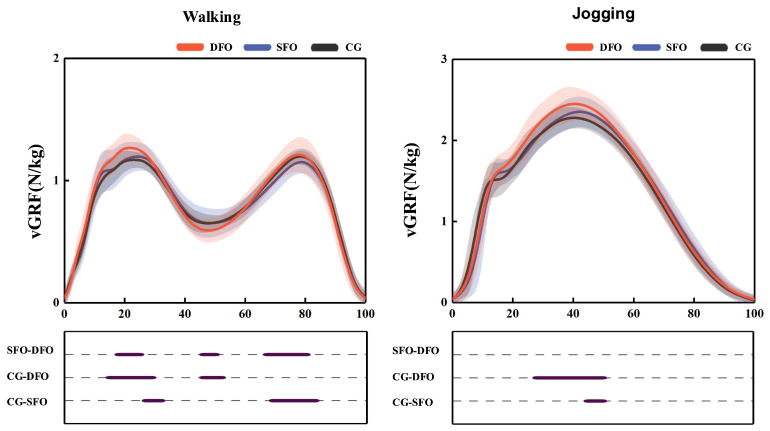
Effects of FO on vGRF during walking and jogging from SPM. DFO represents the condition of wearing shoes with double arch support orthosis, SFO represents the condition of wearing shoes with single arch support orthosis, and CG represents the control condition of not wearing any foot orthoses.

The time-series data for vGRF are shown in Figure 7. During walking, the SFO condition significantly increased vGRF at 29% to 32% of the stance phase (*p* = 0.048) and significantly decreased vGRF at 71% to 83.7% of the stance phase (*p* = 0.041) compared to the control condition. In the DFO condition, vGRF was significantly higher at 17% to 29% (*p* = 0.037) and significantly lower at 48% to 52.9% of the stance phase (*p* = 0.046) compared to the control. When comparing DFO to SFO, vGRF was significantly higher at 19.4% to 24.6% (*p* = 0.039) and significantly lower at 47.1% to 51.6% of the stance phase (*p* < 0.001).

As shown in Table 3, during the early support phase of walking, compared to the control condition, peak vGRF significantly increased by 0.10 N/kg (95% CI: 0.05–0.15, *p* < 0.001) in the DFO condition and by 0.02 N/kg (95% CI: 0.01–0.05, *p* = 0.031) in the SFO condition. During the mid-support phase, the DFO condition significantly reduced the minimum vGRF by 0.09 N/kg (95% CI: 0.05–0.15, *p* < 0.001), while in the late support phase, the SFO condition significantly reduced peak vGRF at toe-off by 0.05 N/kg (95% CI: 0.01–0.08, *p* = 0.009) compared to control.

As shown in Table 4, during jogging, compared to the control condition, the DFO significantly increased peak vGRF by 0.20 N/kg (95% CI: 0.10–0.26, *p* < 0.001), and the SFO significantly increased peak vGRF by 0.08 N/kg (95% CI: 0.01–0.15, *p* = 0.030). Additionally, peak vGRF was considerably greater in the DFO condition than in the SFO by 0.10 N/kg (95% CI: 0.05–0.15, *p* = 0.010).

#### 3.2.2. Lower Limb Moments

**Figure 8:**
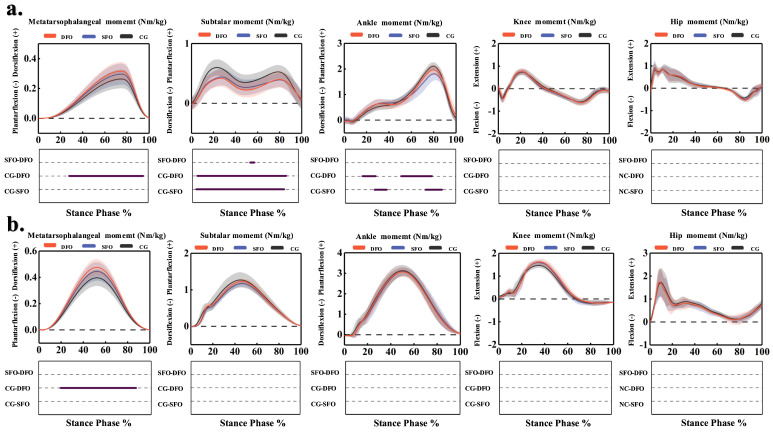
Impact of FO on lower limb joint moments in the sagittal plane during walking and jogging from SPM. (**a**) Walking. (**b**) Jogging. DFO represents the condition of wearing shoes with double arch support orthosis, SFO represents the condition of wearing shoes with single arch support orthosis, and CG represents the control condition of not wearing any foot orthosis.

The time-series data for joint moments are shown in Figure 8a. During walking, SFO significantly reduced subtalar plantarflexion moments from 5.1% to 89.5% of the stance phases (*p* < 0.001) while increasing ankle plantarflexion moments at 28.5% to 37.6% (*p* < 0.001) and decreasing them at 74.5% to 87.7% (*p* < 0.001) of the stance phase. In the DFO condition, metatarsophalangeal joint plantarflexion moments increased significantly from 28.6% to 95% of the stance phase (*p* < 0.001), while subtalar plantarflexion moments decreased significantly from 5.1% to 89.4% (*p* < 0.001). Ankle plantarflexion moments increased at 17.2% to 28.5% (*p* = 0.048) and decreased at 52% to 78.8% of the stance phase (*p* < 0.001). During jogging, only DFO significantly increased metatarsophalangeal joint plantarflexion moments from 20% to 86.8% of the stance phase (*p* < 0.001) compared to the control condition (Figure 8b).

As shown in Table 3, during walking, compared to the control condition, both SFO and DFO significantly increased peak metatarsophalangeal joint plantarflexion moments by 0.03 Nm/kg (95% CI: 0.01–0.06, *p* = 0.002) and 0.05 Nm/kg (95% CI: 0.03–0.07, *p* < 0.001), respectively, significantly increased peak ankle, and significantly decreased peak subtalar joint plantarflexion moments by 0.16 Nm/kg (95% CI: 0.13–0.20, *p* < 0.001) in SFO and 0.18 Nm/kg (95% CI: 0.15–0.21, *p* < 0.001) in DFO. Compared to SFO, DFO significantly increased peak metatarsophalangeal joint plantarflexion moments by 0.02 Nm/kg (95% CI: 0.01–0.05, *p* = 0.004).

As shown in Table 4, during jogging, compared to the control condition, both SFO and DFO significantly increased peak metatarsophalangeal joint plantarflexion moments by 0.05 Nm/kg (95% CI: 0.02–0.08, *p* = 0.004) and 0.08 Nm/kg (95% CI: 0.05–0.11, *p* < 0.001), respectively. Compared to SFO, DFO further significantly increased peak metatarsophalangeal joint plantarflexion moments by 0.03 Nm/kg (95% CI: 0.02–0.04, *p* < 0.001).

## 4. Discussion

We compared the sagittal plane biomechanics of lower limb joints during walking and jogging in females with flexible flat feet while wearing three types of footwear: regular shoes, SFO, or DFO. The experimental results supported the hypothesis: Dual vs. single transverse arch foot orthosis is more effective in improving gait biomechanics in healthy females with flexible flatfoot.

We used high-speed DFIS to characterize foot motion dynamics during gait in healthy females with flexible flat feet. We found that both SFO and DFO significantly increased NTNH and decreased MAA during the stance phase, indicating improved arch support function with both orthoses. These results align with the findings of Hsu et al. [45], but differ from those reported by Ho et al. [30]. The discrepancy may be attributed to variations in the type and material of the orthoses, as well as differences in measurement techniques. Ho et al. used motion capture with reflective markers to quantify arch parameters, which may introduce soft tissue artifacts due to the relative motion between markers and bone, thereby affecting measurement accuracy [30]. In contrast, high-speed DFIS overcomes the limitations of traditional biomechanical motion capture methods, providing a more accurate quantification of foot dynamics during walking [46]. Compared to SFO, the DFO provided superior structural stability, particularly during the mid and late stages of the support phase. This enhanced stability is likely due to the additional support provided to the transverse arch. By reinforcing both the medial longitudinal arch and transverse arch, the DFO enhances the foot’s overall structural integrity, allowing for more effective distribution of plantar pressures and greater resistance to deformation during dynamic activities. Specifically, DFO’s impact on normalizing the NTNH and MAA suggests that it prevents excessive arch collapse by maintaining transverse arch curvature throughout the stance phase [34]. This stability promotes a more even plantar pressure distribution between medial and lateral foot regions, reducing stress on the medial longitudinal arch and mitigating pronation tendencies.

Both the SFO and DFO vs. normal shoe significantly influenced the distribution of vGRF in the stance phase of gait across various stages of the support phase. During walking, SFO increased the initial peak of vGRF at the beginning of the support phase and decreased it toward the end, aligning with previous findings [27]. Similarly, DFO elevated the early vGRF peak but was more effective in reducing vGRF at midstance. Reducing vGRF during midstance helps distribute weight more evenly across the foot, minimizing strain on the medial longitudinal arch and limiting overpronation, thereby enhancing stability. Lower vGRF also decreases the load on the ankle and subtalar joints, reducing compensatory movements and promoting optimal joint alignment. This reduction lessens the muscle effort needed for balance, preventing fatigue and fostering a consistent, stable gait. Additionally, smoother force transitions from midstance to push-off enhance propulsion, making each step more efficient and reducing the risk of instability. Overall, lower midstance vGRF improves foot mechanics, providing greater stability, especially in individuals with flexible flatfoot [10]. The increased initial vGRF peak observed with both orthoses may be due to their structural stiffness, while the reduction in late-phase vGRF with SFO may result from diminished muscle activation [30]. During jogging, both SFO and DFO increased peak vGRF at the end of the support phase, with DFO showing a more pronounced effect. This suggests that DFO enhances propulsion during the toe-off phase, increasing lower limb power output and overall performance in individuals with flat feet. These findings imply that while both orthoses offer benefits, DFO provides superior control over vGRF, highlighting the importance of transverse arch support in optimizing foot dynamics and stability.

The study results demonstrated that both SFO and DFO significantly influenced sagittal plane motion in the metatarsophalangeal, subtalar, and ankle joints during walking and jogging. Both orthoses reduced the dorsiflexion angle of the metatarsophalangeal joint and increased the dorsiflexion angle of the subtalar joint during the early and late support phases. The altered subtalar joint motion was more pronounced with the DFO, placing the joint in a more dorsiflexed and valgus position throughout the gait cycle. This valgus positioning optimizes the muscle force and lever arm of the ankle joint, contributing to better joint mechanics [47,48].

Clinically, individuals with flat feet are at a higher risk of developing plantar fasciitis [49]. The observed reduction in metatarsophalangeal joint dorsiflexion helps limit excessive plantar fascia stretching, thereby lowering the risk of inflammation [50]. During jogging, the DFO showed a more substantial reduction in metatarsophalangeal dorsiflexion, likely due to its dual-arch support, which provides enhanced arch stability under dynamic loading conditions. In the ankle joint, both FOs increased the maximum plantarflexion angle during the load-response phase and decreased the maximum dorsiflexion angle during the push-off phase, consistent with previous studies [48,51,52]. This alteration in ankle motion can be attributed to the stabilizing effect of the orthoses, which control excessive joint movement, reduce unwanted vibrations, and alleviate stress on the ankle joint [53,54]. Meanwhile, the increased knee flexion angle observed with the DFO during the load-response phase may represent a compensatory adjustment for the modified movement patterns of the distal joints.

Generally, although both orthoses improved gait mechanics, the DFO provided superior regulation of lower limb motion, particularly during high-intensity activities such as jogging. This suggests that DFO may offer greater potential in reducing the risk of sports-related injuries compared to SFO during strenuous exercise.

The results suggest that both SFO and DFO significantly reduced plantarflexion moments at the subtalar and ankle joints during the stance phase of walking, consistent with previous studies [30,51]; reduced joint moments in the foot are often associated with a lower risk of overuse injuries [55]. Interestingly, this decrease in joint moments does not compromise forward propulsion in patients with flexible flat feet. Instead, we believe that FOs optimize lower limb movement patterns, enhancing gait efficiency. This is supported by the observation that FOs did not decrease subjects’ self-selected walking speed but led to an increase in speed. In contrast to the SFO, the DFO significantly increased the plantarflexion moment at the metatarsophalangeal joints during the push-off phase, indicating that the transverse arch support provided by DFO not only enhances arch height but also promotes effective pressure distribution and mechanical balance in the lower limbs during dynamic weight-bearing activities. This adjustment helps flat-footed individuals achieve better propulsion and forward momentum. Such improvements have positive implications for daily activities and athletic performance, particularly during prolonged or high-intensity weight-bearing tasks. The enhanced load distribution provided by DFO may reduce fatigue and discomfort in the lower limbs, offering greater support and stability for individuals with flat feet.

Overall, the DFO showed significant advantages in improving arch height, medial arch angle, vertical ground reaction forces, and lower extremity joint kinematics and dynamics in patients with flexible flatfoot. These findings suggest that the dual-arch support structure of the DFO provides more comprehensive biomechanical adjustment and support compared to the SFO, making it particularly beneficial for patients engaged in prolonged gait activities or high-intensity exercise. Furthermore, the substantial improvements in vGRF, joint angles, and joint moments observed with DFO indicate its potential to reduce joint stress and lower the risk of sports-related injuries during daily activities in individuals with flat feet.

This study has several limitations. The small sample size and inclusion of only female subjects limit the generalizability of the findings. Future research should consider larger sample sizes that include. Because sex- and age-related changes in joint mobility, muscle strength, and foot structure can affect orthotic performance, examining the interaction between orthosis type, sex, and age is relevant. Such interactions may suggest that alternative orthosis designs are needed according to sex and age. It is also reasonable to assume that body mass interacts differentially with the compression properties of orthoses and the stiffness of foot arches; hence, the use of SFO and DFO might change with body mass. Furthermore, the results reflect only the immediate effects of different orthoses on flexible flatfoot. Longitudinal studies are needed to confirm the lasting benefits of DFO vs. SFO we observed in the present study acutely. Given the significant effects of DFO on lower extremity joint dynamics, further studies should also investigate its influence on joint motion in the coronal and transverse planes. Previous studies have shown that flatfoot patients exhibit notable alterations in lower extremity biomechanics in these planes, which may be relevant to understanding the full scope of DFO’s impact.

## 5. Conclusions

Dual vs. single transverse arch foot orthosis is more effective in improving gait biomechanics in females with flexible flatfoot. Longitudinal studies are needed to confirm these benefits.

## Figures and Tables

**Table 1:** Parameters of foot orthoses.

Variables	Value
Surface material	Sponge
Support material	Thermoplastic polyurethane
Inner support height (mm)	28
Outer support height (mm)	20
Transverse arch support height (mm)	13.5

**Table 2:** Participants’ descriptive characteristics.

Variables	Mean ± SD
Age (years)	28.0 5.0
Height (cm)	162.0 4.0
Weight (kg)	56.2 3.2
Walking (m/s), CG	1.2 0.1
Walking (m/s), SFO	1.3 0.1
Walking (m/s), DFO	1.3 0.2
Jogging (m/s), CG	2.5 0.1
Jogging (m/s), SFO	2.6 0.1
Jogging (m/s), DFO	2.7 0.1

**Table 3:** Descriptive statistics of various parameters during the stance phase of walking wearing shoes under different conditions.

Variables								Results					
		Mean ± SD						CG vs. SFO		CG vs. DFO		SFO vs. DFO	
		CG		SFO		DFO		95% Confidence Interval	*p*	95% Confidence Interval	*p*	95% Confidence Interval	*p*
MAA	Maximum angle (°)	152.49	1.43	150.24	1.43	147.96	1.87	(−2.56, −1.96)	**<0.001**	(−4.85, −4.22)	**<0.001**	(−2.71, −1.84)	**<0.001**
	ROM (°)	9.22	0.48	8.69	0.81	8.51	0.90	(0.06, 0.99)	0.300	(0.27, 1.14)	**0.003**	(−0.47, 0.83)	0.573
NTNH	Minimum	0.11	0.01	0.12	0.01	0.13	0.01	(−0.01, −0.01)	**<0.001**	(−0.02, −0.02)	**<0.001**	(−0.01, −0.01)	**<0.001**
	ROM	0.07	0.002	0.06	0.01	0.05	0.01	(0.01, 0.02)	**<0.001**	(0.02, 0.03)	**<0.001**	(0.01, 0.02)	**0.005**
vGRF (N/kg)	First peak	1.17	0.03	1.19	0.06	1.27	0.06	(−0.05, −0.01)	**0.031**	(−0.15, −0.05)	**<0.001**	(−0.12, −0.02)	**0.008**
	Second peak	1.20	0.03	1.15	0.03	1.21	0.09	(0.01, 0.08)	**0.009**	(−0.07, 0.05)	0.608	(−0.12, −0.1)	**0.045**
MTPJ	Maximum angle (°)	29.92	7.34	26.47	5.29	23.88	4.25	(0.94, 5.95)	0.08	(3.41, 8.66)	**<0.001**	(1.55, 3.67)	**<0.001**
	ROM (°)	22.27	5.75	19.77	4.05	17.83	3.19	(0.54, 4.44)	**0.013**	(1.01, 6.45)	**<0.001**	(1.14, 2.75)	**<0.001**
	Peak moment (Nm/kg)	0.26	0.06	0.29	0.06	0.31	0.06	(−0.06, −0.01)	**0.002**	(−0.07, −0.03)	**<0.001**	(−0.05, −0.04)	**0.004**
Subtalar	Maximum angle (°)	10.43	1.40	14.46	1.37	14.69	2.27	(−5.17, −2.89)	**<0.001**	(−6.49, −2.02)	**0.002**	(−2.04, 1.58)	0.784
	ROM (°)	21.08	1.83	16.29	1.92	16.91	1.71	(3.17, 6.41)	**<0.001**	(2.53, 5.79)	**<0.001**	(−2.63, 1.38)	0.501
	Peak moment (Nm/kg)	0.61	0.02	0.45	0.04	0.431	0.04	(0.13, 0.20)	**<0.001**	(0.153, 0.213)	**<0.001**	(−0.02, 0.06)	0.300
Ankle	Maximum angle (°)	12.83	1.79	9.25	2.71	8.86	1.67	(1.66, 5.51)	**0.002**	(2.73, 5.22)	**<0.001**	(−0.95,1.73)	0.551
	ROM (°)	24.55	2.75	21.26	3.68	20.70	2.13	(−0.17, 6.75)	0.061	(2.20, 5.50)	**<0.001**	(−2.6, 3.76)	0.710
	Peak moment (Nm/kg)	2.10	0.15	1.83	0.14	1.88	0.09	(0.14, 0.41)	**<0.001**	(0.13, 0.30)	**<0.001**	(−0.166, 0.05)	0.276
Knee	Maximum angle (°)	47.44	6.24	47.17	6.24	45.37	5.51	(−4.33, 4.86)	0.90	(−2.20, 6.35)	0.320	(−1.24, 4.85)	0.228
	ROM (°)	39.45	6.71	39.80	6.81	36.46	5.55	(−5.44, 4.76)	0.90	(−1.60, 7.32)	0.194	(−0.41, 6.81)	0.079
	Peak moment (Nm/kg)	0.75	0.04	0.76	0.08	0.76	0.06	(−0.07, 0.50)	0.73	(−0.05, 0.02)	0.288	(−0.07, 0.06)	0.848
Hip	Maximum angle (°)	29.02	1.44	40.42	2.66	30.22	3.22	(−2.81, −0.22)	0.057	(−0.59, 0.16)	0.080	(−0.94, 1.35)	0.717
	ROM (°)	46.12	3.09	43.91	5.28	45.27	4.92	(−0.36, 4.77)	0.09	(−1.74, 3.43)	0.503	(−3.36, 0.64)	0.171
	Peak moment (Nm/kg)	0.84	0.15	0.88	0.16	0.86	0.10	(−0.20, 0.11)	0.56	(−0.14, 0.11)	0.804	(−0.07, 0.15)	0.488

Note: The bold in the table indicate statistically significant differences, with a significance level set at *p* < 0.05. Values are mean ± SD. CG: control group; SFO: single-arch foot orthosis; DFO: dual-arch foot orthosis; MAA: medial arch angle; NTNH: normalized truncated navicular height; vGRF: vertical ground reaction force; MTPJ: metatarsophalangeal joint; ROM: range of motion.

**Table 4:** Descriptive statistics of various parameters during the stance phase of jogging wearing shoes under different conditions.

**Variables**								**Results**					
		Mean ± SD						CG vs. SFO		CG vs. DFO		SFO vs. DFO	
		CG		SFO		DFO		95% Confidence Interval	*p*	95% Confidence Interval	*p*	95% Confidence Interval	*p*
vGRF (N/kg)	Maximum	2.28	0.13	2.34	0.19	2.45	0.27	(−0.15, −0.01)	**0.030**	(−0.26, −0.10)	**<0.001**	(−0.15, −0.46)	**0.010**
MTPJ	Maximum angle (°)	17.90	4.10	14.97	3.16	13.50	2.58	(1.55, 4.38)	**<0.001**	(2.97, 5.89)	**<0.001**	(0.89, 2.04)	**<0.001**
	ROM (°)	10.14	2.28	8.28	1.77	7.46	1.42	(1.08, 2.66)	**<0.001**	(1.88, 3.48)	**<0.001**	(0.49, 1.15)	**<0.001**
	Peak moment (Nm/kg)	0.40	0.09	0.45	0.10	0.48	0.09	(−0.08, −0.02)	**0.004**	(−0.11, −0.05)	**<0.001**	(−0.05, −0.01)	**<0.001**
Subtalar	Maximum angle (°)	11.46	1.35	13.19	1.38	13.98	1.44	(−2.76, −0.71)	**0.040**	(−3.92, −1.12)	**0.020**	(−1.86, 0.28)	0.131
	ROM (°)	12.59	1.93	10.78	1.68	11.31	2.05	(−0.13, 3.74)	0.064	(−0.44, 2.94)	0.131	(−2.34, 1.23)	0.503
	Peak moment (Nm/kg)	1.28	0.21	1.18	0.08	1.24	0.07	(−0.05, 0.25)	0.173	(−0.11, 0.20)	0.546	(−0.13, 0.01)	0.107
Ankle	Maximum angle (°)	22.93	2.09	20.21	3.38	21.03	4.23	(1.04, 4.39)	**0.004**	(0.15, 3.65)	**0.036**	(−2.59, 0.95)	0.336
	ROM (°)	40.02	6.32	34.76	7.11	38.07	5.67	(0.92, 9.59)	**0.021**	(−0.54, 4.44)	0.114	(−6.86, 0.24)	0.065
	Peak moment (Nm/kg)	3.14	0.15	3.09	0.12	3.07	0.155	(−0.03, 0.11)	0.237	(−0.17, 0.142)	0.119	(−0.05, 0.10)	0.560
Knee	Maximum angle (°)	49.86	3.73	49.40	1.92	49.32	1.44	(−0.72, 1.65)	0.427	(−0.76, 1.84)	0.402	(−0.57, 0.72)	0.818
	ROM (°)	29.81	4.63	30.80	3.02	31.34	2.39	(−2.38, 0.39)	0.154	(−3.21, 0.15)	0.072	(−1.79, 0.72)	0.388
	Peak moment (Nm/kg)	1.62	0.20	1.66	0.14	1.67	0.15	(−0.13, 0.05)	0.358	(−0.11, 0.04)	0.31	(0.04, 0.07)	0.789
Hip	Maximum angle (°)	29.98	5.14	30.77	3.96	30.86	3.68	(−2.97, 1.38)	0.461	(−2.82, 1.07)	0.366	(−0.11, 0.92)	0.868
	ROM (°)	39.03	5.62	39.08	5.34	39.87	4.63	(−2.61, 2.59)	0.996	(−3.31, 1.73)	0.525	(−2.01, 0.44)	0.201
	Peak moment (Nm/kg)	1.99	0.26	1.99	0.42	2.05	0.35	(−0.39, 0.41)	0.959	(−0.40, 0.28)	0.715	(-0.32, 0.18)	0.567

Note: The bold in the table indicate statistically significant differences, with a significance level set at *p* < 0.05. Values are mean ± SD. CG: control group; SFO: single-arch foot orthosis; DFO: dual-arch foot orthosis; vGRF: vertical ground reaction force; MTPJ: metatarsophalangeal joint; ROM: range of motion.

## Data Availability

The data that support the findings of this study are available on reasonable request from the corresponding author. The date is not publicly available, due to privacy or ethical restrictions.

## Abbreviations

The following abbreviations are used in this manuscript:

SFO	single-arch foot orthoses
DFO	dual-arch foot orthoses
ROM	range of motion
FOs	foot orthoses
DFIS	dual fluoroscopic imaging system
vGRF	vertical ground reaction force
NTNH	normalized truncated navicular height
MAA	medial arch angle
SD	standard deviation
